# Efficacy of ozone therapy on visual evoked potentials in diabetic patients

**DOI:** 10.1186/s13098-023-01114-w

**Published:** 2023-06-26

**Authors:** Morteza Izadi, Mohammad Javanbakht, Ali Sarafzadeh, Behzad Einollahi, Farzaneh Futuhi, Zahra Vahedi, Shi Zhao, Nematollah Jonaidi-Jafari, Mahboobeh Sadat Hosseini, Javad Hosseini Nejad, Effat Naeimi, Seyed Hassan Saadat, Hadi Esmaeili Gouvarchin Ghaleh, Mozhgan Fazel, Zahra Einollahi, Luca Cegolon

**Affiliations:** 1 Health Research Center, Baqiyatallah University of Medical Sciences, Tehran, Iran; 2 Nephrology and Urology Research Center, Clinical Science Institute, Baqiyatallah University of Medical Sciences, Tehran, Iran; 3 Department of Biostatistics, Faculty of Medicine, Guilan University of Medical Sciences, Rasht, Iran; 4 Nephrology Department, Loghman Hakim Hospital, Shahid Beheshti University of Medical Sciences, Tehran, Iran; 5 Department of Pediatrics, School of Medicine, Hazrat-E Ali. Asghar Pediatrics Hospital, Iran University of Medical Sciences, Tehran, Iran; 6 JC School of Public Health and Primary Care, Chinese University of Hong Kong, Hong Kong, China; 7 Health Research Center, Lifestyle Institute, Baqiyatallah University of Medical Sciences, Tehran, Iran; 8 Neuroscience Research Center, Baqiyatallah University of Medical Sciences, Tehran, Iran; 9 Endocrinology and Metabolism Department, Baqiyatallah University of Medical Sciences, Tehran, Iran; 10 Behavioral Sciences Research Center, Lifestyle Institute, Baqiyatallah University of Medical Sciences, Tehran, Iran; 11 Applied Virology Research Center, Baqiyatallah University of Medical Sciences, Tehran, Iran; 12 Ozone CRC, BMSU, Tehran, Iran; 13 Nephrology Research Center, Shariati Hospital, Tehran University of Medical Sciences, Tehran, Iran; 14 Department of Medical, Surgical & Health Sciences, University of Trieste, Trieste, Italy; 15 Occupational Medicine Unit, University Health Agency Giuliano-ISontina (ASUGI), Trieste, Italy

**Keywords:** Diabetes mellitus, HbA_1c_, Ozone therapy, Visual evoked potentials

## Abstract

**Background:**

The involvement of the central nervous system is a frequent yet underestimated complication of diabetes mellitus. Visual evoked potentials (VEP) are a simple, sensitive, and noninvasive method for detecting early alterations in central optic pathways. The objective of this paralleled randomized controlled trial was to evaluate the impact of ozone therapy on visual pathways in diabetic patients.

**Methods:**

Sixty patients with type 2 diabetes visiting clinics of Baqiyatallah university in Tehran (Iran) hospital were randomly assigned to two experimental groups: Group 1 (N = 30) undergoing a cycle of 20 sessions of systemic oxygen-ozone therapy in addition to standard therapy for metabolic control; Group 2 (N = 30)—serving as control—receiving only standard therapy against diabetes. The primary study endpoints were two VEP parameters; P100 wave latency and P100 amplitude at 3 months. Moreover, HbA_1c_ levels were measured before the start of treatment and three months later as secondary study endpoint.

**Results:**

All 60 patients completed the clinical trial. P100 latency significantly reduced at 3 months since baseline. No correlation was found between repeated measures of P100 wave latency and HbA_1c_ (Pearson’s r = 0.169, p = 0.291). There was no significant difference between baseline values and repeated measures of P100 wave amplitude over time in either group. No adverse effects were recorded.

**Conclusions:**

Ozone therapy improved the conduction of impulses in optic pathways of diabetic patients. The improved glycemic control following ozone therpay may not fully explain the reduction of P100 wave latency though; other mechanistic effects of ozone may be involved.

## Background

Diabetes Mellitus (DM) encompasses a cluster of common metabolic disorders that cause hyperglycemia. The estimated worldwide prevalence of DM has increased substantially over the past three decades, from 30 million cases in 1985 to 425 million in 2017. The International Diabetes Federation has predicted that if the current trend continues more than 629 million people will develope diabetes by 2045 [1].

DM and its subsequent pathophysiologic changes affecting multiple organs place a heavy burden not only on patients but also on the entire health care systems [2]. Central nervous system disorders are frequent yet underestimated complications of DM [3–7] with diabetic neuropathy being the most common sequela.

For years, retinopathy sustained by vascular pathology was assumed to be the underlying cause of visual dysfunction in diabetic patients. However, diabetes also affects neuronal cells of the retina, resulting in dysfunction and degeneration of some retinal neurons [8].

The main causative factor for early functional changes in diabetic neuropathy and reduced nerve conduction velocity (NCV) is likely to be endoneurial hypoxia [9]. On the other hand, oxidation of elevated levels of intracellular glucose increases the generation of reactive oxygen species (ROS) [10, 11]. Both latter mechanisms—endoneurial hypoxia and ROS generation—determines excessive oxidative stress [12]. Oxidative stress, combined with derangements in vascular and metabolic pathways, contributes to the development of diabetic neuropathy [13].

In experimental studies, treatment with antioxidants [14] proved to restore normal blood flow in diabetic neuropathy, improving NCV and retrieving impaired nerve function [15–21].

Although ozone molecule in large doses is a powerful oxidant and inhalation of ozone gas is very toxic for the lungs [22], recent studies have shown that administration of small doses of a gas mixture of 2% O_3_ plus 98% O_2_ via appropriate routes paradoxically induces an adaptive reaction reducing the endogenous oxidative stress [23–26].

The upregulation of the anti-oxidant system induced by ozone therapy reportedly reduces chronic oxidative stress, thereby improving blood circulation, oxygen delivery to ischemic tissues, insulin secretion and efficacy, inducing also a state of euphoria and wellbeing [23]. For instance, ozone therapy in patients affected by diabetic foot induced normalisation of organic peroxides levels, activation of superoxide dismutase, prevention of oxidative stress and improvement of glycemic control [27]. Furthermore, ozone treatment also reduced the oxidative damage on proteins and lipids of patients affected by multiple sclerosis [28].

Visual evoked potentials (VEP) are electrical potential differences, generated in response to visual stimuli, that can be recorded from the human scalp. VEP are a simple, sensitive, non-invasive and harmless method for detecting early alterations in central visual pathways. Since damage to the optic pathway reduces the amplitude and increases the latency of the response wave. VEPs have become a routine method to diagnose reversal patterns in several neurologic diseases affecting the optic pathway [29–31].

Several studies have found abnormalities of VEP parameters in diabetic patients, especially prolongation of P100 wave latency [29, 32–40]. VEP can detect preclinical neuro-degenerative or micro-vascular changes within or downstream the retina, even in patients without diabetic retinopathy [41]. These changes denote a loss of neuronal function even before the detection of anatomical abnormalities [42].

In view of the above, this randomized controlled study aims to evaluate the effect of ozone therapy to imptove the function of visual pathways in diabetic patients treated at Baqiyatallah University of Medical Sciecnes in Tehran (Iran).

## Methods

This was a single-centre, randomized, controlled, parallel-group study enrolling 60 patients with type 2 diabetes, conducted from May  2019 to February 2020 at the Ozone Complementary Research Center in Tehran, Iran.

In absence of reference estimates, hypothesizing a 25% difference in P100 wave latency between patients treated with ozone as compared to those receiving standard of care for diabetes, assuming a two-sided test with a 5% significance level and a desired 80% statistical power, at least 52 patients (26 patients treated with ozone versus 26 controls) were required to achieve statistically significant results. We therefore decided to slightly increase the latter numbers, recuiting 60 patients (30 per study arm).

SPSS software was used to assign participants to the two experimental groups with an allocation ratio of 1:1, by blind block randomization:

- **Group 1 (treatment arm)** underwent a cycle of systemic oxygen-ozone therapy in addition to standard diabetes therapy for metabolic control.
- **Group 2 (control arm)** did not receive any treatments other than standard diabetes therapy for metabolic control.

Baseline clinical and demographic characteristics of patients, including height, weight and duration of diabetes, were recorded. Patients underwent baseline ophthalmologic examination to classify their retinopathy status as follows:

- No apparent retinopathy,
- Mild non-proliferative diabetic retinopathy (NPDR),
- Moderate NPDR,
- Severe NPDR,
- Regressed Proliferative Diabetic Retinopathy (PDR),
- PDR.

VEP were recorded as primary endpoint, at baseline and after 1, 2 and 3 months. HbA_1c_ levels were used as the secondary outcome and measured at baseline and after 3 months.

No information on side effects was collected.

Exclusion criteria included:

- G6PD deficiency;
- Pregnancy;
- Nursing patients;
- Abnormal coagulation tests;
- Abnormal thyroid function tests;
- Hypersensitivity to ozone; and
- Reduced visual acuity not correctable by glasses.

### Oxygen ozone therapy

Oxygen ozone therapy (major and minor autohemotherapy) was performed twice a week for 20 sessions, and 10 weeks in total. The procedure was as follows

#### Major autohemotherapy

Blood was drawn (1.2 mL/kg) from an antecubital vein into a sterile glass bottle containing citrate sodium (3.8% 10 mL per 100 mL of blood) as anticoagulant. After disconnecting the bottle, a saline infusion was used to keep the vein open. An oxygen-ozone mixture with ozone concentration of 25–30 µg/mL was then added to the blood bottle, which was gently rotated for 5 min to mix the gas blend with blood. The hyper oxygenated and ozonated blood was thereafter reinfused via the same vein over the course of 20 min. The entire procedure required 40 min to complete.

Ozone was produced by Herrmann Medozon compact (Germany). This device measures ozone concentration photometrically in real-time, in accordance with the rules of the International Ozone Association.

#### Minor autohemotherapy

Blood (10 mL) was removed i.v. and collected into a 20 mL disposable syringe prefilled with the same amount of ozone-oxygen mixture (10 mL). The syringe was shaken for 30 s and slowly injected intramuscularly (i.m.).

### Visual evoked potentials

Pattern-reversal VEP was selected as the type of visual stimuli for recording since it is less variable in timing and waveform than VEP elicited by other stimuli. The pattern-reversal stimulus consisted of a checkerboard with 8 × 8 black and white checks (medium-sized 0.5, 30 min of arc) changing phase (from white to black and vice versa) abruptly with a frequency of twice per second (2/s). The patient, sitting comfortably, was placed in front of the checkerboard pattern, at 1 m distance between the eyes and the screen. Patients with refractive errors were tested with appropriate corrective glasses. Patients were asked to focus at the red coloured marker, located at the centre of the checkerboard pattern, with only one eye at time (monocular stimulation). Surface electrodes were employed to record the electrical impulses generated by the patient’s visual pathway in response to pattern alterations.

The surface electrodes were placed with electrode paste after proper skin preparation by cleansing, degreasing and abrading. According to the international 10/20 system, surface electrodes were placed according to bony landmarks and size of the head [43, 44]. The active electrode was placed on the scalp over the occiput and above the inion (Oz), at a distance to inion equal to 10% of the distance between inion (Oz) and nasion. The reference electrode was placed on the forehead at Fz, a point above nasion at a distance to nasion equal to 30% of the distance between nasion and inion. The ground electrode was attached to a nonspecific point, usually the forehead (Fpz). The subscript z indicates a midline position. The difference between impulses received by the active and reference electrodes was used for recording VEP (Oz–Fz).

VEP parameters recorded were P100 wave latency (in ms) and P100 wave amplitude (in µV). P100 wave amplitude was calculated using the peak-to-peak amplitudes of waves N70-P100.

### Statistical analyses

SPSS Version 25 was used to perform the statistical analyses.

VEP parameters of the subjects’s right eyes were used for statistical analysis.

Quantitative variables were expressed as mean ± standard deviation (SD), whereas. categorical variables were expressed as number and percentages (%).

Independent samples t-Test were used to compare the baseline characteristics of ozone-treated patients versus controls and against measurements from 50 healthy individuals without diabetes. One-way ANOVA was used to assess the effect of retinopathy status as between-subject factor on VEP parameters of the eye. Repeated ANOVA tests were performed over time (at baseline, after one month, after two months, and at three months). The level of significance was set at 0.05.

## Results

Diabetic patients were recuited from May through November 2019, and the clinical trial ended in February 2020. All 60 patients completed the trial.

### Baseline demographic and clinical characteristics of the studied population

As can be seen from Table 1, there was no difference in the baseline distribution of variables between the two patient groups. Patients’ age ranged between 42 and 82 years, with a mean of 60.8 ± 9.2 years in the treatment arm against 61.7 ± 9.6 years in the control arm. The duration of DM ranged between < 7 days (for newly diagnosed cases) up to 31 years, for a mean of 14.2 ± 6.7 days in ozone-treated patients versus 14.4 ± 6.9 days in controls. The mean baseline HbA_1c_ was 9.04 ± 1.46 days in ozone treated patients against 8.96 ± 1.54 days in controls; 90% of patients had HbA_1c_ > 53 mmol/mol (7.0%). The mean BMI was 27.7 ± 4.7 kg/m^2^ in patients treated with ozone against 27.6 ± 3.9 kg/m^2^ in controls.

**Table 1:** Baseline clinical and demographic characteristics of the studied population

Baseline variables	Treatment arm (N = 30)	Control arm (N = 30)	P-value
Age (years)			
Mean ± SD	60.8 ± 9.2	61.7 ± 9.6	N.S
40–49	5 (16.7)	5 (16.7)	N.S
50–59	8 (26.67)	7 (23.3)	
60–69	11 (36.7)	12 (40)	
70–79	5 (16.7)	4 (13.3)	
80+	1 (3.3)	2 (6.7)	
Sex			
Male	17 (56.7)	16 (53.3)	
Female	13 (43.3)	14 (46.7)	
Duration of DM (days)—(mean ± SD)	14.2 ± 6.7	14.4 ± 6.9	N.S
BMI (kg/m^2^)—(mean ± SD)	27.7 ± 4.7	27.6 ± 3.9	N.S
HbA1C (%)—(mean ± SD	9.04 ± 1.46	8.96 ± 1.54	N.S

*DM* diabetes mellitus, *N.S* non significant

There was no significant difference in baseline retinopathy status between the two patient groups. Of total 60 right eyes, the percentage of retinopathy status was as follows:

- 16.7% no apparent retinopathy;
- 35% mild NPDR;
- 15% moderate NPDR;
- 6.7% severe NPDR; and
- 26.6% regressed PDR.

### Baseline VEP measurements

Table 2 shows the reference data of VEP parameters obtained from 50 non-diabetic volunteers. As can be seen, compared to males, females had lower P100 latency values (p < 0.05). By contrast, no significant difference was found for P100 amplitude by sex in each group.

**Table 2:** Baseline VEP parameters from 50 non-diabetic subjects, diabetic patients treated with ozone and controls, by sex

Group	VEP parameter	Total (mean ± SD)	Males (mean ± SD)	Females (mean ± SD)	P-value
Healthy subjects	P100 wave latency (ms)	98.83 ± 4.48	101.22 ± 4.50	96.60 ± 4.45	0.002
	P100 amplitude (μV)	7.08 ± 2.08	6.54 ± 2.02	7.67 ± 2.09	0.085
Treatment arm	P100 wave latency (ms)	108.33 ± 5.36	111.82 ± 5.37	103.76 ± 5.32	0.001
	P100 amplitude (μV)	4.03 ± 1.89	3.98 ± 1.90	4.09 ± 1.88	0.112
Control arm	P100 wave latency (ms)	108.31 ± 5.29	111.53 ± 5.30	104.68 ± 5.22	0.001
	P100 amplitude (μV)	4.02 ± 1.91	3.96 ± 1.91	4.08 ± 1.90	0.105

As can be seen from Table 2, compared to non-diabetic individuals, diabetic patients treated with ozone had significantly longer baseline P100 wave latency (108.33 ± 5.36 vs. 98.83 ± 4.48 ms; p < 0.001) and lower P100 wave amplitude (4.03 ± 1.89 vs. 7.08 ± 2.08 μV; p < 0.001). By contrast, there was no significant baseline difference in P100 wave latency and amplitude by study arm.

Table 3 shows number and percentage of diabetic patients with abnormal baseline VEP findings by study arm. VEP were considered altered when latency or amplitude of P100 wave differed from normal values by at least two standard deviations.

**Table 3:** Number and percentage of diabetic patients with abnormal baseline VEP findings, by study arm

Parameter	Patient group	Number (%)
P100 wave latency (ms)	Treatment arm	14 (46.6)
	Control arm	14 (46.6)
P100 amplitude (μV)	Treatment arm	6 (20)
	Control arm	5 (16.6)

#### Correlation between baseline VEP parameters and HbA1C or duration of diabetes

There was no significant correlation between baseline measurements of VEP parameters and HbA1C or duration of diabetes.

#### Effect of retinopathy status on baseline VEP parameters

There was no significant correlation between retinopathy status and baseline P100 wave latency [F(5; 54)=0.783; p = 0.566] or amplitude [F(5; 54)=1.76; p= 0.136) in both patient groups.

### Comparison between groups over the time

**Fig. 1:**
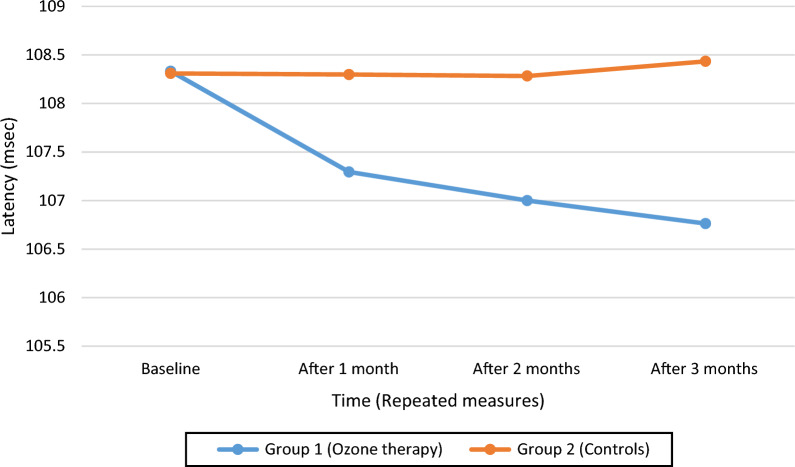
Distribution of P100 wave latency over time, by study arm (ozone-treated patients versus controls)

**Fig. 2:**
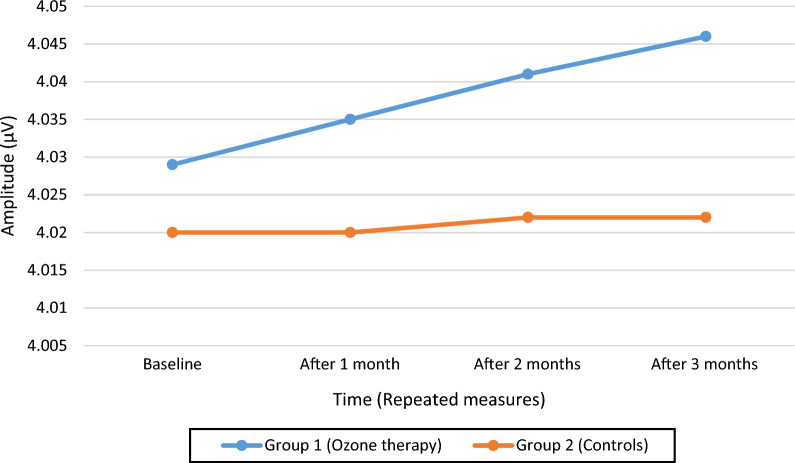
Distribution of P100 wave amplitude over tine, by study arm (ozone-treated patients versus controls)

**Fig. 3:**
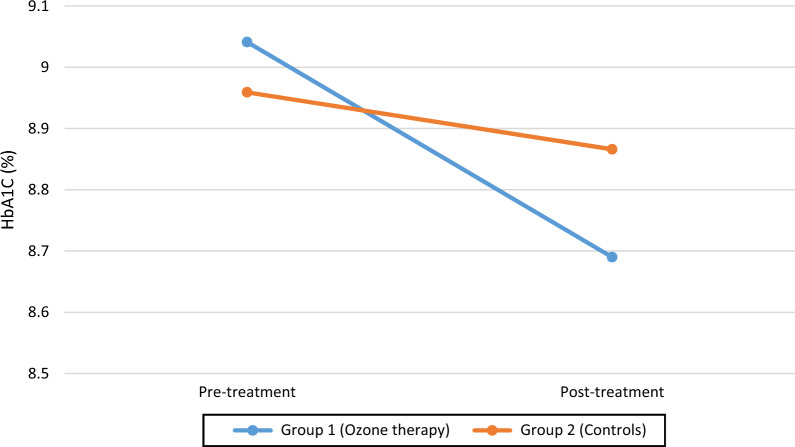
Comparison of HbA_1c_ measurements before (at baseline) and after ozone treatment (at 3 months), by study arm (ozone-treated patients versus controls)

Table 4 shows the variation of VEP parameters (P100 latency and amplitude) as well as HbA1C from baseline to month 3, by patients group and between ozone-treated patients against non-diabetic individuals. Figures 1, 2 and 3 show the trend over time of P100 Latency, P100 Amplitude and HbA1C, respectively, by study arm.

**Table 4:** Comparison of VEP measurements at baseline and after 3 months by patient group (ozone treated diabetic patients versus controls). Comparison of VEP measurements of ozone-treated diabetic patients both at baseline and after 3 months against values from individuals without diabetes

VEP parameter	Timeline	Study arm		P-value*	Non-diabetic subjects	P-value**
		Treatment	Control			
P100 wave latency (ms)	Baseline	108.33 ± 5.36	108.31 ± 5.29	N.S	98.83 ± 4.48	< 0.001
	At 3 months	106.76 ± 5.27	108.43 ± 5.31	0.032		< 0.001
P100 amplitude (μV)	Baseline	4.03 ± 1.89	4.02 ± 1.91	N.S	7.08 ± 2.08	< 0.001
	At 3 months	4.05 ± 1.93	4.02 ± 1.90	N.S		< 0.001
HbA1C (%)	Baseline	9.04 ± 1.46	8.96 ± 1.54	N.S		
	At 3 months	8.69 ± 1.47	8.87 ± 1.63	0.041		

*N.S* non significant

*Comparison of treatment versus control arm

**Comparison of ozone-treated patients with non-diabetic patients

#### P100 latency

As can be seen from Table 4, P100 latency decreased from 108.33 ± 5.36 ms to 106.763 ± 5.27 ms in patients treated with ozone, whereas it slightly increased from 108.31 ± 5.29 ms to 108.434 ± 5.31 ms in controls. The difference in mean P100 latency at 3 months between treatment and control arm was statistically significant (p = 0.03).

As can be seen from Table 4, although the mean P100 latency estimates decreased significantly at 3 months in patients treated with ozone, they were still significantly higher than non-diabetic subjects (p < 0.001).

Since the assumption of sphericity was violated at Mauchly’s test(χ^2^(5) = 392.701, p < 0.001), the degrees of freedom were corrected using Greenhouse–Geisser estimates of sphericity (ε = 0.387). Whilst the individual effect of time [F(1.16; 67.33)=3.24; p=007] or patient group [F(1; 58)=0.53; p=0.47] was not significant, there was a significant time × group interaction [F(1.16; 67.33) = 3.84; p = 0.048] with an effect size f(U) = 0.20.

#### P100 amplitude

As can be seen from Table 4, P100 amplitude slightly increased from 4.03 ± 1.89 μV to 4.05 ± 1.93 μV in diabetic patients treated with ozone, wheres it remained stable (4.02 μV) in controls. However, the difference in mean P100 amplitude at 3 months between treatment and control arm was not significant

Since the assumption of sphericity was violated at Mauchly’s test (χ^2^(5) = 50.583, p < 0.001), the degrees of freedom were corrected using Greenhouse–Geisser estimates of sphericity (ε = 0.715). Both the individual effect of time [F(2.14; 124.41) = 1.16, p = 0.310] or patient group [F(1; 58) = 0.004; p = 0.952] was non-significant. The interaction term of Time × Group was also not significant [F(2.14; 124.41) = 0.581; p = 0.572 with an effect size f(U) = 0.27.

There was no significant difference between baseline values of P100 amplitude and their repeated measures over time in either study group (Fig. 2).

#### HbA1C

As can be seen from Table 4, in patients treated with ozone HbA1C decreased from 9.04 ± 1.46 % to 8.69 ± 1.47 % after 3 months. Among controls, HbA1C diminshed from 8.96 ± 1.54 % to 8.87 ± 1.63 % at month 3. Reduction of mean HbA1C at 3 months was significantly stronger in patients treated with ozone compared to controls (p = 0.041). Whilst there was a significant main effect of time [F(1; 58) = 23.16; (p < 0.001], the effect of patient group was non-significant, [F(1.58)=0.022; p = 0.882]. However, a significant ime × group interaction could be appreciated [F(1; 58) = 7.54;  p = 0.008].

No correlation was observed between HbA1C measurements and variaton of P100 wave latency over time (Pearson’s r = 0.169; p = 0.291).

### Side effects

No relevant adverse effects were noted in either group.

## Discussion

### Key findings

In the present study conduction of impulses across optic pathways, in terms of reduction of P100 latency, significantly improved 3 months since start of ozone therapy, entailing a cycle of 20 treatment sessions. However, no significant difference in P100 wave amplitude was observed from baseline through month 3.

No correlation was found between P100 wave latency or amplitude and duration of diabetes or HbA1 levels.

Finally, the degree of diabetic retinopathy did not correlate with abnormal VEP in the present study.

### Interpretation of findings

Stimulation of the central region of the visual field leads to generation of P100 waves, mostly occurring in the striate cortex [45]. The number of functional afferent fibres reaching the striate cortex determines the amplitude of VEP [46]. A reduction in baseline P100 wave amplitude of diabetic patients observed in our study is in line with the open literature [29, 47–49]. Although ozone therapy had a noticeable impact on P100 wave amplitude, the respective effect size was not significant, probably due to small number of treatment sessions or participants.

Albeit ozone therapy was previously found to significantly reduce fasting blood sugar (FBS) level in diabetic patients [50], the reduction in P100 wave latencies observed in the present study could not be merely explained by the effects of the gas on glycemic control. Ozone in fact mitigates the oxidative stress mainly by shifting the balance of endogenous oxidant-antioxidant systems towards anti-oxidantion, another potential mechanism for therapeutic effects of ozone [51]. As already mentioned above, oxidative stress enhances the risk of developing neuropathy in diabetic patients with micro-angiopathy [13]

Retinopathy was not associated with VEP abnormalities in the present trial a finding in line with other studies [39, 52–55]. However, further investigations reported prolonged VEP latencies only in patients with proliferative retinopathy [56–59], Whilst most neurophysiologic changes in diabetic patients can be attributed to ischemic damage to retinal neurons and other structures induced by microangiopathy, other factors may also play an key role in diabetic neuropathy.

Several studies investigated the relationship between P100 latency changes and long-term glycemic control, with conflicting evidence though [33, 37, 40, 60, 61]. Whilst correlation between P100 latency changes and long-term glycemic control (expressed as HbA1C levels or glycaemia) was reported by a few studies [33, 34, 60], several others did not confirm the latter association [29, 35, 38, 59, 62–66].

For instance, baseline P100 wave latencies of 30 newly diagnosed diabetic patients with mean HbA1C levels of 79.2 mmol/mol were significantly longer than healthy age- and sex-matched controls’ (p < 0.01), whereaes N75 to P100 amplitudes were similar between the two groups. Six months later, when all diabetic patients achieved glycemic control (mean HbA_1c_ = 55.2 mmol/mol), all VEPs parameters were completely normalised [34].

By contrast, in a study on 18 non-insulin-dependent diabetic patients contrasted with 35 normal controls at baseline and after 4.6 years, VEP alterations remained stable over time (at least 4 years) without correlating with metabolic control during the study period. Conversely, peripheral neurological disease progressed in the latter study, correlating positively with metabolic control [32].

In another study on 12 diabetic patients with poor glycemic control, VEPs were recorded before and 3+ days after treatment with continuous subcutaneous insulin infusion leading almost to normoglycemia. Four diabetic patients (33.3%) had abnormal baseline VEPs. In comparison with controls, diabetic patients had longer mean P100 wave latencies (p < 0.01). Three days after close control of blood glucose, leading to a significant fall in the mean level of blood glucose (from 13.77 ± 2.2 mmol/L to 6.8 ± 1.2 mmol/L  p < 0.01), a significant reduction in the mean P100 wave latencies (112.5 ± 7.6 ms; p < 0.01) was observed. Nevertheless, compared with normal values, P100 wave latencies in diabetic patients were still significantly longer, with no correlation between VEPs improvement and fall in blood glucose [38]. Likewise, P100 wave latencies in diabetic patients were significantly longer than individuals' without diabetes in the present study, a finding largely consistent with the open literature [4, 29, 44, 45, 47].

By providing information on pathways distal from the optic nerve, pattern electroretinography (PERG) enables to distinguish VEP delays due to optic nerve disorders from those arising from downstream pathways [67]. An index of neural conduction in the retinocortical pathway can be created by comparing peak implicit times of PERG and VEPs [68, 69]. In a study measuring both VEP and pattern electroretinogram, a proportion of diabetic patients showed abnormal VEP latencies in absence of fundoscopic findings of retinopathy, suggesting impaired retinal function and in some cases optic neuropathy [70]. Given abnormal VEP parameters in diabetic patients could be due to retinal or optic tract disorders or both, future research should use both PERG and VEP simultaneously, to better isolate the area on visual pathways targeted by ozone therapy.

### Study limitations

A relatively short duration of follow-up was the main limitation of this study. Further studies with a larger sample size should be conducted to confirm whether ozone therapy can also increase the amplitude of the P100 wave. Extended follow-ups would enalble to clarify the timeline for P100 wave parameters to return to pre-treatment levels since cessation of ozone therapy.

Furthermore, the present study was conducted with a single electrode placed over the midline of occiput which is called a 1-channel VEP. This central electrode picks up signals from the combined hemispheres at the visual cortex. Another option is to use three occipital electrodes for a 3-channel VEP. With 3-channel electrode placement it is possible to detect optic nerve misrouting and determine whether a lesion is located at or posterior to the chiasm.

## Conclusion

Ozone therapy reduced P100 wave latency and improved glycemic control in diabetic patients. Ozone therapy could be recommeded as a complementary treatment alongside standard therapy to improve the conduction of impulses in visual pathways of diabetic patients. A treatment cycle of ozone therapy should probably entailed at least 20 sesssions.

The beneficial effect of ozone needs to be explored more in depth though, to understand potential further therapeutic mechanims against diabetic neuropathy beyond glycemic control. Given abnormal VEP parameters in diabetic patients could be due to retinal or optic tract disorders or both, simultaneous PERG and VEP are recommended in future research studies, to isolate the area on visual pathways targeted by ozone therapy.

## Data Availability

The datasets generated during or analysed during the current study are available from the corresponding author on reasonable request.
