# Correction: Efficacy of ozone therapy on visual evoked potentials in diabetic patients

**DOI:** 10.1186/s13098-023-01127-5

**Published:** 2023-07-07

**Authors:** Morteza Izadi, Mohammad Javanbakht, Ali Sarafzadeh, Behzad Einollahi, Farzaneh Futuhi, Zahra Vahedi, Shi Zhao, Nematollah Jonaidi‑Jafari, Mahboobeh Sadat Hosseini, Javad Hosseini Nejad, Effat Naeimi, Seyed Hassan Saadat, Hadi Esmaeili Gouvarchin Ghaleh, Mozhgan Fazel, Zahra Einollahi, Luca Cegolon

**Affiliations:** 1 Health Research Center, Baqiyatallah University of Medical Sciences, Tehran, Iran; 2 Nephrology and Urology Research Center, Clinical Science Institute, Baqiyatallah University of Medical Sciences, Tehran, Iran; 3 Department of Biostatistics, Faculty of Medicine, Guilan University of Medical Sciences, Rasht, Iran; 4 Nephrology Department, Loghman Hakim Hospital, Shahid Beheshti University of Medical Sciences, Tehran, Iran; 5 Department of Pediatrics, School of Medicine, Hazrat-E Ali. Asghar Pediatrics Hospital, Iran University of Medical Sciences, Tehran, Iran; 6 JC School of Public Health and Primary Care, Chinese University of Hong Kong, Hong Kong, China; 7 Health Research Center, Lifestyle Institute, Baqiyatallah University of Medical Sciences, Tehran, Iran; 8 Neuroscience Research Center, Baqiyatallah University of Medical Sciences, Tehran, Iran; 9 Endocrinology and Metabolism Department, Baqiyatallah University of Medical Sciences, Tehran, Iran; 10 Behavioral Sciences Research Center, Lifestyle Institute, Baqiyatallah University of Medical Sciences, Tehran, Iran; 11 Applied Virology Research Center, Baqiyatallah University of Medical Sciences, Tehran, Iran; 12 Ozone CRC, BMSU, Tehran, Iran; 13 Nephrology Research Center, Shariati Hospital, Tehran University of Medical Sciences, Tehran, Iran; 14 Department of Medical, Surgical & Health Sciences, University of Trieste, Trieste, Italy; 15 Occupational Medicine Unit, University Health Agency Giuliano-ISontina (ASUGI), Trieste, Italy

**Correction** **:** **Diabetology & Metabolic Syndrome (2023) 15:140** **https://doi.org/10.1186/s13098-023-01114-w**

Following the publication of the original article [1], it was noted that due to a typesetting error the affiliation of both the authors were wrongly placed. The correct affiliations are provided in this correction and the original article has been corrected.

## References

[1] Izadi M, Javanbakht M, Sarafzadeh A, Einollahi B, Futuhi F, Vahedi Z, Zhao S, Jonaidi-Jafari N, Hosseini MS, Nejad JH, Naeimi E, Saadat SH, Ghaleh HEG, Fazel M, Einollahi Z, Cegolon L (2023). Efficacy of ozone therapy on visual evoked potentials in diabetic patients. Diabetol Metab Syndr.

